# Integrated Pleiotropic Gene Set Unveils Comorbidity Insights across Digestive Cancers and Other Diseases

**DOI:** 10.3390/genes15040478

**Published:** 2024-04-10

**Authors:** Xinnan Wu, Guangwen Luo, Zhaonian Dong, Wen Zheng, Gengjie Jia

**Affiliations:** 1 Institute of Public-Safety and Big Data, College of Data Science, Taiyuan University of Technology, University Street, Yuci District, Jinzhong 030600, China; wuxinnan1527@link.tyut.edu.cn; 2 Shenzhen Branch, Guangdong Laboratory of Lingnan Modern Agriculture, Genome Analysis Laboratory of the Ministry of Agriculture and Rural Affairs, Agricultural Genomics Institute at Shenzhen, Chinese Academy of Agricultural Sciences, Shenzhen 518120, China; luoguangwen@caas.cn (G.L.); dongzhaonian@caas.cn (Z.D.)

**Keywords:** digestive cancers, comorbidity, pleiotropic gene set, protein-protein interaction, heterogeneity in genetic predisposition, immunity-related function

## Abstract

Comorbidities are prevalent in digestive cancers, intensifying patient discomfort and complicating prognosis. Identifying potential comorbidities and investigating their genetic connections in a systemic manner prove to be instrumental in averting additional health challenges during digestive cancer management. Here, we investigated 150 diseases across 18 categories by collecting and integrating various factors related to disease comorbidity, such as disease-associated SNPs or genes from sources like MalaCards, GWAS Catalog and UK Biobank. Through this extensive analysis, we have established an integrated pleiotropic gene set comprising 548 genes in total. Particularly, there enclosed the genes encoding major histocompatibility complex or related to antigen presentation. Additionally, we have unveiled patterns in protein-protein interactions and key hub genes/proteins including *TP53*, *KRAS*, *CTNNB1* and *PIK3CA*, which may elucidate the co-occurrence of digestive cancers with certain diseases. These findings provide valuable insights into the molecular origins of comorbidity, offering potential avenues for patient stratification and the development of targeted therapies in clinical trials.

## 1. Introduction

Cancer stands as a leading cause of death worldwide, posing a significant obstacle to the increase in life expectancy across every country [1]. Among these cancers, digestive cancers are particularly severe, constituting 26% of global cancer incidence but accounting for 35% of all cancer-related deaths [2]. This places a substantial burden on global healthcare systems. The most prevalent digestive cancers include colorectal cancer (with approximately 1.15 million new cases in 2020), esophageal cancer (0.60 million new cases), liver cancer (0.91 million new cases), pancreatic cancer (0.50 million new cases) and stomach cancer (1.09 million new cases) [3]. As we continue to confront the challenges posed by cancer, there is an escalating demand for clinical healthcare to address the management of cancer patients who are coping with multiple concurrent diseases, commonly referred to as comorbidities. This is increasingly recognized as the new norm in cancer care [4]. Numerous studies have demonstrated that patients with digestive cancers frequently present with or develop a range of complex diseases that can significantly complicate their prognosis. For instance, various gastrointestinal complications, including increased odds ratio or relative risk of esophageal cancer, stomach cancer, liver diseases (including hepatobiliary cancer) and pancreatic cancer, have been associated with obesity [5]. In the case of male hepatocellular carcinoma, diabetes, alcohol-related liver disease and hepatitis C virus infection were identified as major individual comorbidities, with population-attributable fractions exceeding 10% [6]. In China, cardiometabolic diseases, particularly hypertension, were the most common comorbidities of gastric cancer and esophageal cancer, with hypertension being predominant. The proportion of esophageal cancer patients with three or more comorbidities increased from 0.1% to 2.2% from 2010 to 2019 [7]. Diabetes, hyperlipidemia, inflammatory bowel disease and polyps were identified as four types of comorbidities in colorectal cancer [8]. It is important to note that in patients with advanced pancreatic cancer, it is comorbidity, not age, that serves a prognostic factor [9]. Despite the prevalence of comorbidities in digestive cancers, integrated reporting on this topic has been limited.

Exploring the underlying genetic factors can also provide insights into the shared mechanisms of diseases’ comorbidity and aid in the identification of potential drug targets. Multidrug resistance displays a major obstacle to effective therapeutic interventions against cancer. The development of resistance against anticancer agents can be due to individual genetic differences, such as mutations, gene drug-resistant genes expression, altered epigenetics, enhanced drug efflux, cell death inhibition, and various other molecular and cellular mechanisms [10]. Genetic pleiotropy, a phenomenon where a single gene or genetic variant influences multiple traits, appears to be a common occurrence in human genome [11]. A comprehensive analysis by Watanabe et al. [12], which examined over 4000 publicly available Genome-Wide Association Studies (GWAS), revealed widespread pleiotropy at both the gene level (63%) and single nucleotide polymorphism (SNP) level (31%). The identification of potential susceptibility genes and the discovery of pleiotropic effects can help elucidate the reasons behind shared heritability and comorbidity among various complex traits and provide valuable insights into the underlying biological mechanisms governing these traits [11].

However, prior studies have typically focused on individual diseases or a limited set of known health conditions, leaving many diseases under-studied or undetected. Another limitation of the traditional approach is its concentration on examining a small number of genes in isolation, overlooking the intricate interconnectedness of genes through pathways and protein-protein interactions (PPIs). This places a substantial burden on global healthcare systems, particularly in terms of their pleiotropic structures and their impact on outcomes.

The modern era presents us with an abundance of publicly available genetic data resources related to human diseases, offering a unique opportunity to construct an integrated set of pleiotropic genes (genes that directly influence multiple traits [13]) associated with both five digestive cancers and comorbid diseases. We list a few of these exemplary resources here. MalaCards [14], an integrated human disease knowledge base, aggregates annotated disease information from various data sources, including Elite genes (genes presumed to cause diseases) and variations from ClinVar [15] and UniProt [16]. The GWAS Catalog [17], provided by NHGRI-EBI, compiles a reliable database of summary-level information regarding SNP-trait associations in human genome-wide association studies. The UK Biobank [18] (UKB) serves as a large-scale individual-level database, encompassing genetic data, diagnoses, lifestyle information, and many other health-related data from over half a million participants in the United Kingdom. FUMA [19] is a web-based tool platform capable of annotating, prioritizing, visualizing and interpreting GWAS results. EpiGraphDB [20], both a graph database and a tool platform, houses a wide array of biomedical and epidemiological relationships. With the availability of these databases and tools, we shall shift the research paradigm from considering only a handful of co-occurring diseases associated with a few pleiotropic genes to encompassing a broad spectrum of diseases attributed to interacting gene networks. Consequently, methods that enable such research are highly desirable.

Our study seeks to address the three aforementioned issues by developing an integrated analysis workflow that leverages the resources mentioned, allowing us to gain deeper insights into digestive cancers.

## 2. Materials and Methods

### 2.1. Data Sources

We aimed to include as many diseases as possible from a selection of 567 major groups of disease diagnoses [21]. These groups were created based on the unique International Classification of Diseases (ICD) codes, which were organized according to diagnosis records and clinical manifestations. Consequently, we identified a total of 150 diseases that were present in at least one of the following databases: MalaCards and GWAS Catalog, and had corresponding entries in the UK Biobank (UKB) electronic health record (EHR) database. More details about the data sources can be found in the ‘URLs’ section.

### 2.2. Data Analysis Methods

#### 2.2.1. Identification of Comorbid Diseases in Five Digestive Cancers

We conducted an integrated study to identify the presence of comorbid diseases associated with five digestive cancers. This investigation was based on the UKB dataset (Approval ID 78814), and EHRs from a cohort of 458,038 individuals were extracted. We aimed to explore the associations between digestive cancers and other diseases. In this regard, Fisher’s exact tests were performed in the R programming language for each pair of diseases by calculating their co-occurrence within the UKB’s diagnosis records phenotypic data. We considered a disease to be a comorbidity only if the odds ratio was larger than 1, and the false discovery rate (i.e., the *p*-value adjusted through the Benjamini-Hochberg procedure) was less than 0.05 in the Fisher’s exact test.

#### 2.2.2. Pleiotropic Gene Set Construction

We gathered potential susceptibility genes of each disease from five distinct types of data, which were obtained from three primary sources:

(1) **MalaCards Database:** We initiated the process by searching for the disease name on the MalaCards website (refer to URLs) and clicking on the ‘show all’ button for related sections, including ‘Genes’, ‘ClinVar’, and ‘UniProtKB/Swiss-Prot’. We retrieved relevant information that contained in each of these URLs
(2) **GWAS Catalog:** First, we searched each disease’s name on the GWAS Catalog website (see URLs) and downloaded the relevant GWAS Catalog files for each disease (see Table S1). Second, we extracted the genes associated with each disease from these downloaded files. Our selection criteria included a significance threshold $\left(P_{\mathrm{GWAS}}<5\times 10^{-8}\right)$ and manual inspection (excluding unrelated diseases, such as those labelled as ‘measurement’ in the ‘MAPPED TRAIT’ column).
(3) **UKB GWAS Data:** This dataset was curated following GWAS analysis of 7221 phenotypes across six continental ancestry groups in the UKB [22]. Our approach was based on the ‘UKBB GWAS Imputed v3-File Manifest Release 20180731.xlsx’ file (see URLs). Firstly, we queried each disease name in the ‘Description Lookup’ sheet to obtain the ‘phenotype code’ for each disease (see Table S1). Secondly, we downloaded ‘variants.tsv.bgz’ and each ‘<phenotype code>.gwas.imputed v3.both sexes.tsv.bgz’ using the provided commands in the ‘Manifest 201807’ sheet. We then converted variant locations to variant rsids, beta coefficients to odds ratios (OR = exp(beta)) and so on in order to obtain the GWAS summary statistics file in the required FUMA format [23]. Thirdly, we uploaded these GWAS summary statistics to the FUMA ‘SNP2GENE’ website, setting default parameters (such as $P_{\mathrm{GWAS}}<5\times 10^{-8}$), except for specific configurations: Reference panel population: UKB release2b; Minimum Minor Allele Frequency (≥): 0.001; eQTL mapping → Tissue types: Select all; Gene types → Gene type: Protein coding; MAGMA gene expression analysis: Select ‘GTEx v8:54 tissue types’ and ‘GTEx v8:30 general tissue types’. Subsequently, we downloaded the ‘Gene table (mapped genes)’ files, which provided us with the list of genes.

We then created the potential susceptibility gene set for each disease by amalgamating the susceptibility genes from the three aforementioned sources. Finally, we constructed the pleiotropic gene set by identifying overlapped susceptibility genes for each disease pair among the five digestive cancers and the other 145 diseases.

#### 2.2.3. Dendrogram Analyses

To construct dendrogram trees for all 150 diseases, we employed several R functions and tools. Initially, we used the ‘dist’ function in R to compute and generate a distance matrix based on a specified distance measure. This matrix computed the distances between the rows of a data matrix, considering the susceptibility genes associated with all diseases. Subsequently, we applied the ‘hclust’ function in R, which conducts hierarchical cluster analysis using a set of dissimilarities to cluster the objects. The result obtained from the ‘dist’ operation served as the input for ‘hclust’. We further transformed the ‘hclust’ object into a newick file format using the ‘hc2Newick’ function from the ctc R package. This step was pivotal in creating the dendrogram trees for all 150 diseases. To visualize these dendrogram trees, we utilized the iTOL [24] online platform (accessible via URLs). iTOL is an online tool designed for displaying, annotating and managing phylogenetic and other tree-like structures.

#### 2.2.4. Definition of Pleiotropic Structure and Hub Genes/Proteins in Disease Pairs

For evaluating the potential pleiotropy type, we harnessed the R package ‘epigraphdb’ [20,25], which integrates gene-protein connections with comprehensive information about biological pathways and protein-protein interactions (PPIs). Primarily, we employed the querying function ‘query_epigraphdb’ within this package to submit data requests to an EpiGraphDB API endpoint. This function facilitated the mapping of genes to proteins (UniProt [16]) and proteins to the pathways in which they are found, using Reactome [26] data. Subsequently, we extracted information regarding the specific pathways associated with these genes and proteins. In the implementation, we imported the overlapped susceptibility genes for each disease pair.

We established criteria for determining the type of pleiotropy in disease pairs. When a group of proteins linked to a single SNP was mapped to the same biological pathway and/or exhibited protein-protein interactions, we considered it more likely that the SNP operated through vertical pleiotropy [20]. If these proteins were involved in the same biological pathway and/or possessed PPIs, we categorized the disease pair as exhibiting vertical pleiotropy; otherwise, we categorized it as exhibiting horizontal pleiotropy.

In network analysis, a hub node is characterized by having a high degree of edges, indicating that it interacts with numerous other nodes in the network [27]. Therefore, if the protein(s) shared the most pathways and PPIs in a disease pair, we identified the protein(s) and their corresponding gene(s) as the hub protein(s)/gene(s) for that disease pair.

#### 2.2.5. Functional Enrichment Analysis

In our functional enrichment analysis, we aimed to assess the enrichment levels of pleiotropic susceptibility genes for each disease pair in various biologically relevant categories. This included the Gene Ontology (GO) gene sets, consisting of 10,532 items, which were obtained for this study through the use of the ‘msigdbr’ function from the ‘msigdbr’ R package. To determine the statistical significance of the enrichment, we employed the hypergeometric distribution, utilizing the ‘phyper’ function from the ‘stats’ R package and calculating *p*-values, following the equation below:

(1) p-value=∑i=knniN−nK−iNK

In this equation, binom *N* represents the sample size, which corresponds to the total number of genes considered in the enrichment analysis; *K* is the number of pleiotropic susceptibility genes associated with the disease pair; *n* is the number of genes associated with the reference gene set, and; *k* denotes the number of correct predictions, indicating the number of genes in the set *K* that also appear in the reference set *n*. When assessing the entire gene sets for enrichment, the estimated significance level was adjusted to account for multiple hypothesis testing. Thus q-values were calculated using the Benjamini-Hochberg procedure, ensuring a robust evaluation of the statistical significance of enrichment in different biologically relevant categories.

### 2.3. URLs

Here are the URLs for the various resources mentioned in the text: 

MalaCards: http://www.malacards.org/ (accessed on 1 March 2024).

EpiGraphDB: https://epigraphdb.org/ (accessed on 1 March 2024).

GWAS Catalog: https://www.ebi.ac.uk/gwas/ (accessed on 1 March 2024).

UK Biobank: https://www.ukbiobank.ac.uk/ (accessed on 1 March 2024).

FUMA: https://fuma.ctglab.nl (accessed on 1 March 2024).

UKBB GWAS Imputed v3: https://docs.google.com/spreadsheets/d/1kvPoupSzsSFBNSztMzl04xMoSC3Kcx3CrjVf4yBmE (accessed on 1 March 2024).

## 3. Results

### 3.1. Developing a Workflow to Collect Potential Susceptibility Genes for Five Digestive Cancers and Other Diseases

In order to extensively identify comorbidities for the five digestive cancers, we adopted extensive searches. We referred to total of 567 major groups of disease diagnoses [21], categorized based on ICD unique codes and clinical manifestations. These 567 traits were used as queries across four different databases: MalaCard, GWAS Catalog, UKB GWAS and UKB EHR. Due to variations in disease labeling schemes in these databases, we used the most similar keywords to manually establish mappings between these databases (see Table S1 for detailed query disease names). As a result, we identified a total of 150 diseases that appeared in at least one of the three databases and also existed in the UKB EHR database. These diseases spanned across 18 different disease categories (see Supplementary Table S6 for the detailed information), covering a broad spectrum of the human system. For instance, the Digestive disease category included 11 diseases, which encompassed the five digestive cancers and six other digestive diseases; the Immune disease category included 22 diseases, which is maximum among all the categories, and Metabolic disease category contained 2 diseases. The diverse range of diseases ensured the representativeness of the comorbidity sources for the five digestive cancers.

**Figure 1:**
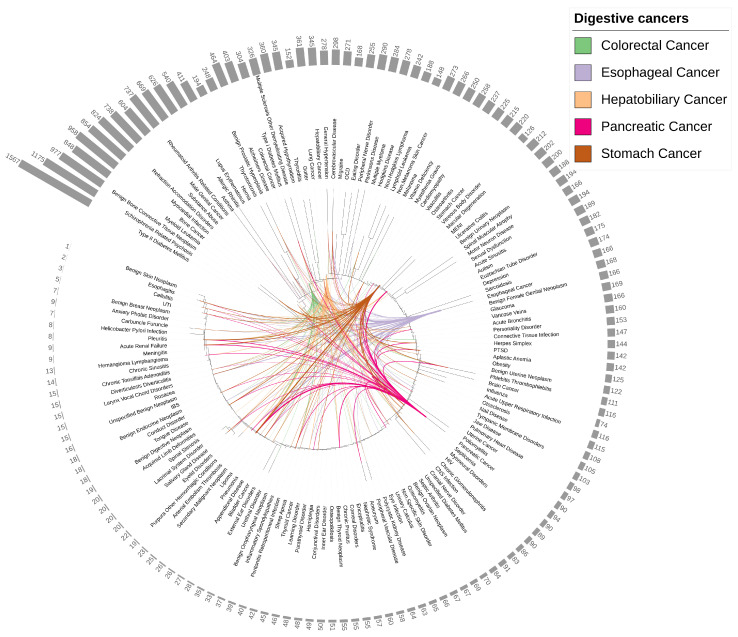
The landscape of the 150 diseases based on overlapped potential susceptibility genes. The 150 diseases were ordered basing on their potential susceptibility genes. The lines between diseases indicate comorbidity relationship of the connected diseases, different colours represent different digestive cancers, the line thickness is proportional to ORlog. The bar height and the number on the outermost layer indicate the number of potential susceptibility genes of each disease.

By amalgamating data from five different sources of genetic data (elite, ClinVar, UniProtKB, GWAS Catalog and UKB GWAS) and from three sources database (Malacard, GWAS Catalog and UKBB GWAS), we compiled the susceptibility genes for each of the 150 diseases (see Table S3). The dendrogram in Figure 1 depicted a landscape of the similarities between these 150 diseases based on their susceptibility genes. Regarding the count of susceptibility genes, frequently-researched diseases generally exhibited the highest number of susceptibility genes. For example, Type II Diabetes Mellitus possessed the most susceptibility genes (1567 genes), followed by Schizophrenia Related Psychosis (1175 genes), Benign Bone Connective Tissue Neoplasm (977 genes) and Bone Cancer (958 genes). In contrast, rarely-researched diseases harbored fewer susceptibility genes, with Urinary Tract Infection (UTI) having only 5 genes, Cellulitis with 3 genes, Esophagitis with 2 genes, and Benign Skin Neoplasm with the fewest at just 1 gene.

In terms of dendrogram similarity, it was observed that shared susceptibility genes formed the basis of similarity between diseases. This was evident in the dendrogram displayed in Figure 1 and Table S4, where: (I) Among the five digestive cancers, Pancreatic Cancer was the most distant from the other four, with Stomach Cancer in the middle. Colorectal Cancer and Hepatobiliary Cancer were closer to each other, sharing as many as 21 susceptibility genes, greater than any other cancer pairs (see Figure S2). (II) For Colorectal Cancer, Thyroid-related diseases, including Goiter (12 shared genes), Thyroiditis (16 shared genes) and Acquired Hypothyroidism (23 shared genes), were closely related to it, as well as Type I Diabetes Mellitus (18 shared genes), Multiple Sclerosis Other Demyelinating Disease (19 shared genes) and Lung Cancer (44 shared genes). (III) For Hepatobiliary Cancer, cardiovascular and cerebrovascular diseases, including General Hypertension (13 shared genes), Cerebrovascular Disease (7 shared genes), Migraine (8 shared genes), Obsessive-Compulsive Disorder (OCD, 4 shared genes), Peripheral Nerve Disorder (7 shared genes) and Parkinson’s Disease (10 shared genes), were very close to it. (IV) For Stomach Cancer, Ophthalmological diseases, including Macular Degeneration (7 shared genes), Vitreous Body Disorder (6 shared genes)) and Multiple Endocrine Neoplasia Type I (MENI, 2 shared genes) were closely related to it. (V) For Esophageal Cancer, Sarcoidosis (6 shared genes) and Benign Female Genital Neoplasm (1 shared gene) were close to it. (VI) For Pancreatic Cancer, Poliomyelitis (6 shared genes) was related to it.

These findings highlight the interconnectedness and shared susceptibility genes between digestive cancers and various comorbid diseases, providing valuable insights into disease relationships and potential comorbidities.

### 3.2. Establishing a Catalogue of Comorbidities for Five Digestive Cancers from EHRs

Understanding the relationships between diseases, such as comorbidities, has significant socio-economic implications, influencing clinical study design and healthcare planning [28]. The presence of comorbidity can markedly alter the clinical symptoms, prognoses and characteristics of diseases. Thus, we investigated these relationships across various disease categories. The logarithm of odds ratio (ORlog) of two diseases represents the strength of their co-occurrence, indicating the degree of comorbidity between each disease pair.

In total, there were 725 (5 × 145) disease pairs formed between the five digestive cancers and the 145 diseases; there were 10 $\left(c_{5}^{2}\right)$ disease pairs within the five digestive cancers themselves, resulting in 735 disease pairs overall. With the threshold of the adjusted *p*-value less than 0.05 and ORlog larger than 0 (i.e., OR > 1), we identified a total of 251 comorbidity pairs out of 735 pairs (34.15%). These comorbidity pairs involved 85 diseases out of the 150 studied (57.33%). As shown in Figure 1 and Table S2, the comorbidities of each digestive cancer were dispersed across diverse disease categories. Twenty-five diseases were shared comorbidities among all five digestive cancers, including Acute Renal Failure, Cerebrovascular Disease, Myocardial Infarction, General Hypertension, Lung Cancer, and Diabetes Mellitus, among others (a complete list of comorbid diseases can be found in Figure S1). Conversely, some diseases, such as Acquired Hypothyroidism, Allergic Rhinitis and Autism, were not comorbid with any of the digestive cancers.

Each of the five digestive cancers had its own set of comorbidities. For example, Colorectal Cancer had 64 comorbidities, with the highest number in the Immune disease category (9 comorbidities), followed by the Cardiovascular disease category (8 comorbidities). Esophageal Cancer had 49 comorbidities in total, with the highest numbers in the Immune and Cardiovascular disease categories (both with 7 comorbidities), followed by the Digestive disease category (6 comorbidities). Hepatobiliary Cancer had a total of 52 comorbid diseases, with the highest numbers in the Immune and Cardiovascular disease categories (both with 8 comorbidities), followed by the Digestive disease category (7 comorbidities). Pancreatic Cancer had 44 comorbidities, primarily in the Immune disease category (8 comorbidities), followed by the Cardiovascular disease category (7 comorbidities). Stomach Cancer had a total of 51 comorbidities, spread across the Immune, Digestive and Infectious disease categories (all of the three with 7 comorbidities).

As illustrated in Figure S1, Stomach Cancer and Esophageal Cancer exhibited a similar pattern of comorbidity consistency, as did Hepatobiliary Cancer and Pancreatic Cancer. In contrast, Colorectal Cancer displayed a distinct profile of comorbidities, including conditions like Cardiomyopathy, Benign Ovarian Neoplasm, Goiter, Uterine Cancer and Urethral Disorder.

**Figure 2:**
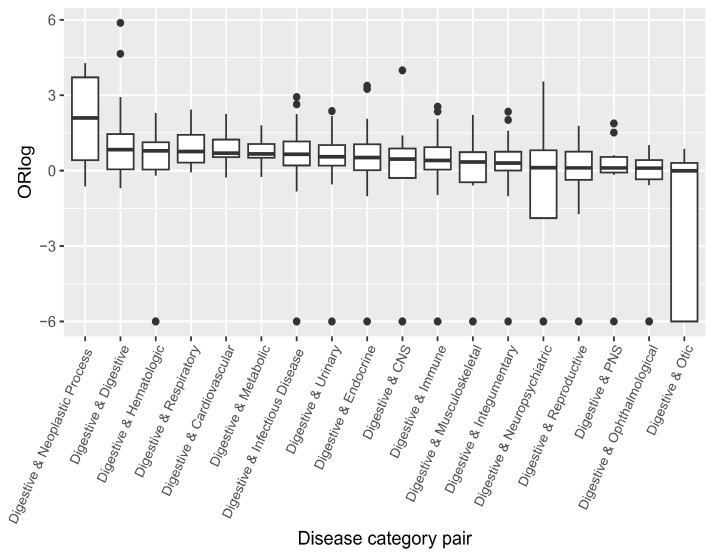
Odds ratio distribution between digestive category and other categories. Each dot represents ORlog value between any one disease from digestive category and any one disease from other disease category. The distribution is in a descending order of the median of ORlog between each disease of digestive category and each disease of other categories. ORlog was set to ‘−6’ if ORlog eq ‘−Inf’. Each category’s diseases are listed in Supplementary Table S6.

At the disease category level, as illustrated in Figure 2, the most likely comorbid category of the Digestive disease category was Neoplastic Process with the highest ORlog median value. It was not a surprise since the five digestive cancers are considered neoplastic diseases. Additionally, the Digestive disease category showed a strong tendency for comorbidity within the category itself. The top comorbid categories for the Digestive disease category were Hematologic, Respiratory, Cardiovascular, Metabolic and Infectious disease, suggesting that these diseases may be susceptibility comorbidities for the five digestive cancers and should receive increased attention in healthcare planning.

Our findings also aligned with known comorbidity patterns reported in the medical community, such as Colorectal Cancer and Diverticulosis Diverticulitis [29], Esophageal Cancer and Obesity [30], Hepatobiliary Cancer and HIV [31], Pancreatic Cancer and Type II Diabetes Mellitus [32], and Stomach Cancer and Cerebrovascular Disease [33]. These reports corroborated our results, confirming the clinical relevance of the identified comorbidities. For example, complications associated with Stomach Cancer included bleeding, perforation and pyloric stenosis, with bleeding being the most common issue requiring modern surgery [34,35]. Colorectal Cancer was linked to complications such as obstruction, perforation, abscess formation, acute appendicitis, ischemic colitis and intussusception [36]. Patients with Hepatobiliary Cancer often had underlying chronic liver disease, cirrhosis, chronic kidney disease and end-stage renal disease, and tumor rupture was a potentially life-threatening complication [37,38,39]. Thrombosis was often linked to Pancreatic Cancer, with a prevalence of thromboembolism in Pancreatic Cancer patients as high as 60% at autopsy compared to 15–25% in other malignancies [40]. Moreover, diabetes associated with Pancreatic Cancer was often diagnosed concomitantly with the cancer or within two years before the cancer diagnosis [41].

### 3.3. Identifying Integrated Pleiotropic Genes and Pleiotropic Structures between Five Digestive Cancers and 145 Diseases

Among the 251 comorbid disease pairs found in the catalogue of comorbidity of the five digestive cancers, 175 pairs (69.7%) shared overlapped genes. For the remaining 484 (735 − 251) non-comorbidity disease pairs, 338 pairs (69.8%) exhibited overlapped genes. By combining the genes shared among the 175 comorbidity pairs and 338 non-comorbidity disease pairs, we created an integrated pleiotropic gene set within and between the five digestive cancers and the other 145 diseases. Overall, this gene set encompassed 548 pleiotropic genes (see Figure S2), with Colorectal Cancer and Hepatobiliary Cancer having the highest number of pleiotropic genes (21 genes).

Colorectal Cancer had 52 comorbidities with pleiotropic genes, with the highest number in the Cardiovascular disease category (8 comorbidities), followed by the Immune disease category (7 comorbidities). Esophageal Cancer had 35 comorbidities with pleiotropic genes, with the highest number in the Cardiovascular disease category (6 comorbidities), followed by the Immune category (4 comorbidities). Hepatobiliary Cancer had a total of 41 comorbidities with pleiotropic genes, with the highest number in the Immune category (7 comorbidities) and the Cardiovascular category (7 comorbidities), followed by the Digestive category (5 comorbidities). Pancreatic Cancer had 19 comorbidities with pleiotropic genes in total, with the highest number in the Cardiovascular category (4 comorbidities) and the Immune category (4 comorbidities). Stomach Cancer had 37 comorbidities with pleiotropic genes, with the highest number in the Digestive category (6 comorbidities), followed by the Immune category (4 comorbidities) and the Infectious disease category (4 comorbidities).

Regarding the 175 comorbidity disease pairs, 74 pairs (42.3%) exhibited horizontal pleiotropic structures (as defined in the Methods section), while 101 pairs (57.7%) displayed vertical pleiotropic structures after an analysis of shared pathways and PPIs (see Table S4). Notably, as illustrated in Figure S3, more than 50% of the comorbidity disease pairs for Colorectal Cancer and Hepatobiliary Cancer exhibited vertical pleiotropic structures, suggesting that vertical pleiotropy plays a dominating role in these two digestive cancers and distinguishes them from the other three digestive cancers.

In order to find out hub genes that may underlie the co-occurrence between a digestive cancer and other diseases, we examined the combined frequencies of each protein in shared pathways and PPIs (see Methods). As presented in Table S4, we identified 33 hub proteins between Colorectal Cancer and other diseases, with P01911 (encoded by the *HLA-DRB1* gene) being the most frequently occurring protein (7 times). There were 27 hub proteins between Esophageal Cancer and other diseases. There were 29 hub proteins between Hepatobiliary Cancer and other diseases, with Q14765 (encoded by the *STAT4* gene) being the most frequently occurring protein (4 times). There were 13 hub proteins between Pancreatic Cancer and other diseases, with P04637 (encoded by the *TP53* gene) as the most frequently occurring protein (4 times). There were 19 hub proteins between Stomach Cancer and other diseases, again with P04637 as the most frequently occurring protein (4 times).

Interestingly, in addition to *TP53*, genes like *KRAS*, *CTNNB1* and *PIK3CA* were also found among the high-occurrence hub proteins for the five digestive cancers. *TP53* is a well-known tumor suppressor gene, with mutations found in over half of all human cancers, particularly in the early stage of cancer, playing a crucial role in the carcinogenesis of the digestive tract [42]. *KRAS* is an oncogene, mutated in approximately 35–45% of colorectal cancers [43]. *CTNNB1* is part of a complex of proteins that constitute adherens junctions, essential for the formation and maintenance of epithelial cell layers by regulating cell growth and adhesion. It is a driver gene in stomach cancer [44]. *PIK3CA*, the catalytic subunit of *PI3K*, coordinates a diverse range of cell functions, including proliferation and survival, and is the third most frequently mutated gene in stomach cancer [45]. These findings suggest that these well-known oncogenes and tumor suppressor genes of digestive cancers may drive or mediate pleiotropy between the five digestive cancers and the other 145 diseases.

### 3.4. Correlating the Likelihood of Co-Occurrence and Shared Genetic Factors for Disease Pairs

After establishing a catalogue of comorbidity and an integrated pleiotropic gene set between five digestive cancers and other diseases, we aimed to investigate the associations between comorbidities and hereditary factors. Many studies often characterize disease comorbidity solely based on shared genetic origins, overlooking pathway-based commonalities between diseases [28]. To overcome this limitation, a few studies have aimed to infer disease-disease relationships by considering genetic overlaps, functional overlaps and comorbidity [46]. We, therefore, sought to explore the relationships between comorbidities and their shared genetic factors, not only based on the ratio of overlapped susceptibility genes, but also on the ratios of shared pathways and PPIs.

**Figure 3:**
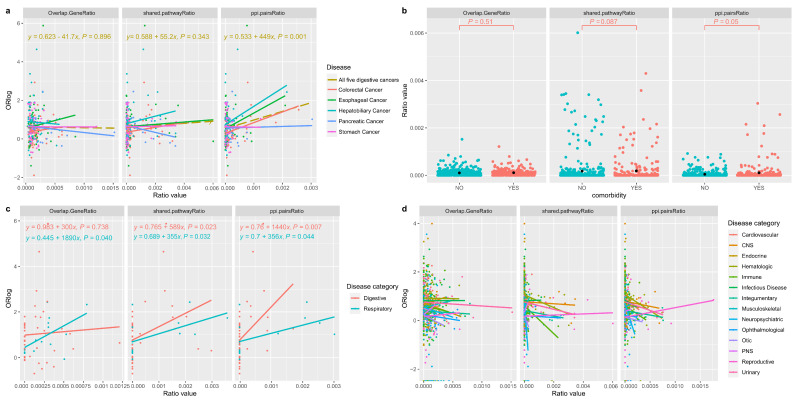
Correlation of odds ratio and overlapped potential susceptibility genes, shared pathways and PPI pairs. Overlap.GeneRatio: OverlapGeneNum/(Disease1Ngene × Disease2Ngene); shared.pathwayRatio: shared_pathwayNum/(Disease1Ngene × Disease2Ngene); ppi.pairsRatio: ppi_pairsNum/(Disease1Ngene × Disease2Ngene). OverlapGene: pleiotropic genes of each disease pair; Disease1Ngene: the number of susceptibility genes for disease 1; Disease2Ngene: the number of susceptibility genes for disease 2; shared_pathway: in each disease pair, for each pair of proteins we match and get the pathways they have in common; ppi_pairs: protein-protein interactions (PPIs) pairs in a disease pair. (**a**) Correlation for disease pairs of vertical pleiotropic structure at three levels (three ratios). (**b**) Statistic differences between comorbid and non-comorbid disease pairs at three levels. ‘YES’ indicates comorbidity disease pairs, ‘NO’ indicates non-comorbidity disease pairs. (**c**) Significant positive correlations for the digestive and respiratory categories at at least one level. (**d**) Non-significant positive correlations for other 14 disease categories at all three levels.

It is important to note that two categories (Metabolic and Neoplastic Process), each containing fewer than 3 diseases, were excluded from this analysis, as they did not provide sufficient data to calculate R^2^ values. Figure 3a illustrates the correlations between logarithm of odds ratio (ORlog) and the ratio of overlapped susceptibility genes, shared pathways ratio and PPIs between five digestive cancers and other diseases. The positive relationships were not statistically significant at the levels of both gene number ratio (*p* = 0.896) and shared pathways ratio (*p* = 0.343), but they became significant at the level of PPIs ratio (*p* = 0.001). Figure 3b demonstrates that the differences between non-comorbidity disease pairs and comorbidity disease pairs were not statistically significant at the levels of both gene number ratio (*p* = 0.51) and shared pathways ratio (*p* = 0.087), but they became significant at the level of PPIs ratio (*p* = 0.05). Both Figure 3a,b indicate that the number of overlapped genes and shared pathways alone may not predict the likelihood of co-occurrence. Instead, the more extensive the interactions (PPIs) between disease pairs, the greater the likelihood of these disease pairs co-occurring. In other words, disease pairs with higher correlations (odds ratios) tended to be exhibit stronger connections within the PPI network. This suggests that PPIs could potentially mediate comorbid relationships between diseases. This finding aligns with the observations made in Carlota et al.’s work [28], where it was revealed that functional overlap contributed to nearly 95% of the associations in the disease network.

In Figure 3c, we see that for the Respiratory disease category (comprising three diseases), the positive correlations were all statistically significant at all three levels (gene number ratio (*p* = 0.04), shared pathways ratio (*p* = 0.032) and PPIs ratio (*p* = 0.044)). Similarly, for the Digestive disease category (with 11 diseases), the positive correlations were statistically significant at both the shared pathways ratio (*p* = 0.023) and PPIs ratio levels (*p* = 0.007). This suggests that comorbidity disease pairs involving diseases in the Respiratory or Digestive categories and other diseases categories were more likely to be genetically linked. For example, certain inherited mutations increase the risk of developing Lung Cancer [47] and Sleep Apnea [48] and about 8% of lung cancer patients have familial risk factors [47,49]. However, in Figure 3d, for the remaining 14 disease categories, none of the relationships were significantly positive at all three levels. This discrepancy may be attributed to the fact that many diseases within these categories lack sufficient susceptibility genes, indicating that not all diseases within each category are primarily genetically driven and promoted. Some diseases might be more influenced by environmental factors, lifestyle or treatment-induced factors [50]. This diversity may reflect disease-category-specific heterogeneity in the hereditary risk factors underlying these conditions.

### 3.5. Unravelling Functional Pathways for Pleiotropic Genes in Disease Pairs between Five Digestive Cancers and 145 Diseases

Although digestive cancers exhibit diverse etiology and the underlying genetic mechanisms are better understood in specific cell and tissue types, there are still shared features among gastrointestinal cancers of different origins. Our study aimed to identify common genes and molecular mechanisms, and to analyze the pleiotropic effects that contribute to the pathogenesis of five digestive cancers. Even though digestive cancers can originate from different organs, they may have some correlations. The presence of complex comorbid diseases can complicate cancer prognosis but may also indicate common pathogenic mechanisms. However, the mechanisms underlying these common links between five digestive cancers and other diseases are not well understood. To address this, we investigated the functional pathways associated with pleiotropic genes using Gene Ontology (GO) gene sets, which include 10,532 gene sets categorized into Molecular Function (MF, 1772 gene sets), Cellular Component (CC, 1009 gene sets) and Biological Process (BP, 7751 gene sets).

**Figure 4:**
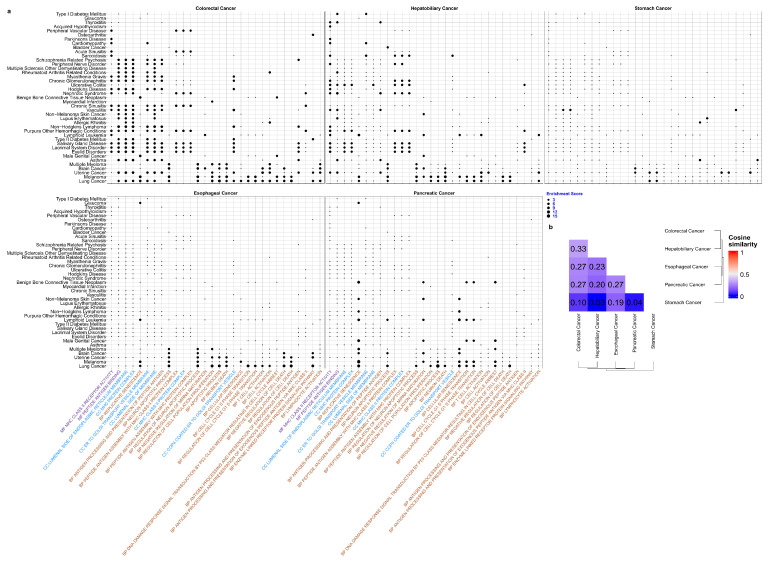
(**a**) Distribution of enrichment in GO gene-sets between five digestive cancers and other diseases. Dots indicate that there are pleiotropic genes for the disease pair and the adjusted *p*-value of enrichment analysis is less than 0.05. Dot size is proportional to enrichment score. X axis only shows the top 30 most-times enriched gene sets, purple colour indicates MF in GO, blue colour indicated CC in GO, and red colour indicates BP in GO. (**b**) Within five digestive cancers cosine similarity based on all 514 enriched GO gene sets’ enrichment score.

As illustrated in Table S5, there were a total of 514 gene sets in which pleiotropic genes shared between digestive cancers and other diseases were significantly enriched. There were 55 diseases in total that had significantly enriched gene sets shared with digestive cancers. Figure 4a shows all 55 diseases on the y-axis and the top 30 enriched gene sets on the x-axis. Among the five digestive cancers, Colorectal Cancer and Hepatobiliary Cancer shared the largest number of functional gene sets, with a cosine similarity of 33% (see Figure 4b). Conversely, Hepatobiliary Cancer and Stomach Cancer shared the smallest number of functional gene sets, with a cosine similarity of 3% (see Figure 4b), in concordance with the shared gene situations previously mentioned.

At the disease level, Lung Cancer exhibited the most instances of enriched gene sets with five digestive cancers, occurring 484 times. This was followed by Melanoma (154 times), Uterine Cancer (138 times), Brain Cancer (73 times) and Multiple Myeloma (48 times). At the gene set level, among the top 30 gene sets, there were two MF gene sets, six CC gene sets and 22 BP gene sets. Notably, gene sets related to the Major Histocompatibility Complex (MHC) (e.g., MF MHC Class II Receptor Activity, CC MHC Protein Complex, BP Peptide Antigen Assembly With MHC Class II Protein Complex) and antigen processing and presentation (e.g., MF Peptide Antigen Binding, BP Antigen Processing And Presentation Of Peptide Antigen, BP Antigen Processing And Presentation Of Endogenous Peptide Antigen) were the most frequently enriched gene sets, appearing six times out of the top 30 gene sets.

In summary, the 55 diseases that share enriched functional gene sets with digestive cancers, as well as the 514 identified gene sets, especially MHC and antigen related ones should be given top priority in future research. By thoroughly investigating the genetic pathogenesis underlying their co-occurrence, a better understanding of their interconnected pathogenetic processes can contribute to more effective disease prevention and early intervention, ultimately leading to better outcomes and prognosis for patients.

## 4. Discussion

Comorbidities driven by pleiotropic genes represent a critical aspect of the complex relationships between digestive cancers and other diseases. These comorbidities can potentially manifest symptoms earlier than the digestive cancers themselves, which typically remain asymptomatic in their early stages. As a result, they can serve as valuable indicators for early detection and diagnosis of digestive cancers. However, It is essential to acknowledge that explaining or predicting comorbidity risks solely based on genetics is challenging, as numerous non-genetic factors, such as diet and lifestyle, play significant roles in disease development. Diet, for instance, is a well-established factor influencing digestive cancers. Diet choices, including the consumption of salty and smoked foods, and low intake of fruits and vegetables can increase the risk of gastric cancer [51], while a diet rich in processed or red meat can increase the risk of colorectal cancer [52]. Recent advances in nutritional management for patients with digestive cancers highlight the role of protein intake modulation in achieving nutritional and clinical benefits. Optimizing both the quantity and quality of protein intake is a potential avenue for improving outcomes for individuals with these cancers [53].

There were streamlined approaches that had general applicability, especially considering the fact of disease co-occurrence. People often reported PPIs [54], shared metabolism [55], and multiple types of input data at the same time [56]. There were a few of studies using topological methods for disease analysis, focusing on one or a few specific diseases [57]. These analyses used EHRs for topological inference, sometimes incorporating the temporal order of diseases in a patient’s history, producing topological disease networks [58]. Another group of studies generated disease networks by computing pairwise, disease-disease correlations or relative risk scores [59,60]. These were inferring correlation instead of causation. We preferred to look for genetic roots, acting mechanism and workable intervention. Statistical approaches to infer causation, such as mendelian randomization (causal inference) coupled with the wealth of data present an opportunity to investigate a working mechanism in depth. Here, this study is deliciated to deeply examine genetic pleiotropy that can drive the co-occurrence between digestive cancers and other diseases.

Our understanding of the genetic players involved in complex traits is currently limited, resulting in what is referred to as “missing heritability” [61]. To address this issue, our approach offers two key contributions. First, we propose extending the analysis to include potential genes through expression quantitative trait loci (eQTL) mapping and to identify new pathways by leveraging known pathway players. Second, we can provide a rationalization of the relationships between genes, thereby enhancing our understanding of their interactions. We applied data mining techniques to the integrated dataset to uncover valuable insights into disease mechanisms and potential interventions with relevance to population health. Our study offers a in-depth examination of genetic pleiotropy and its role in driving the co-occurrence of digestive cancers and other diseases. By analyzing relationships at the genetic level, we provide a foundation for understanding how genes and pathways interact to contribute to disease comorbidity. The strategy proposed here involves targeting drug interventions based on the structure of the disease pair. In cases where the disease pair form horizontal structures, interventions shall be designed to target multiple proteins, whereas vertical structures would involve targeting a single upstream casual protein.

Despite the advantages of our strategy, there are still rooms to be improved. Firstly, the method used to define disease comorbidity based on the associations between diseases could be enhanced to consider sequential occurrences [62]. Additionally, although we were able to expand the gene set through eQTL mapping, our analysis was still constrained by the lack of comprehensive coverage of GWAS data for all diseases. Third, the results of functional enrichment analyses depend on the chosen database and the parameters setting. Different databases may contain different genetic and functional information, which can lead to inconsistencies and biases in the results. Some databases may contain hypothetical or computational gene sets, which can lead to unconfirmed results. Strict parameters setting may filter out some intrinsic functional pathways while lenient parameters setting may introduce artifacts. Fourth, our study was also constrained by the current state of knowledge about pathways and their statistical significance. It is important to note that the biological processes described by pathways leave the possibility of inaccuracy or incompleteness [63]. Therefore, our findings should be considered as meta-analysis, and further validation may be required. Fifth, the complexity of protein participation in various pathways depending on context [64] underscores the importance of confirming the validity of these relationships. Lastly, gene-targeted cancer therapies this study mainly discussed were mainly about chemotherapeutic agents. But most chemotherapeutic agents are related to multidrug resistances [10], while natural compounds produced by living organisms are potential efficient agents for treating cancer without or with much less multidrug resistances, enhancing survival rates and reducing the number of deaths [65]. For example, the therapeutic efficacy of Berberine on colon, pancreatic, liver, and intestine cancers has been reported in several studies. Berberine inhibits cancer cell proliferation by lowering epithelial-mesenchymal transition protein expression, or inducing apoptosis and regulating the cell cycle as well as autophagy, or hinders cancer cell invasion and metastasis by down-regulating metastasis-related proteins [66]. So, natural compounds agents are also needed to be considered into gene-targeted cancer therapies.

## 5. Conclusions

This study has provided an integrated data resource, encompassing an integrated collection of susceptibility genes and a pleiotropic gene set containing 548 genes associated with five digestive cancers and 145 other diseases. We found disease-category-specific heterogeneity in terms of hereditary risk factors by correlating co-occurrence likelihood and genetic factors for disease pairs. In addition, we identified specific functions such as MHC or antigen-related gene sets, patterns of PPIs and hub genes/proteins (e.g., *TP53*, *KRAS*, *CTNNB1* and *PIK3CA*) that may underlie the comorbidity between digestive cancers and other diseases. These molecular insights shed light on how comorbidity may initiate at the molecular level. We also compared the similarities among five digestive cancers in terms of potential susceptibility genes, comorbidities, pleiotropic gene structures and enrichment analyses. We aimed to identify mechanistic pathways underlying disease pathogenesis, actionable targets, and potential intervention strategies. Additionally, this study unveiled multiple pleiotropic structures associated with the co-occurrence of complex diseases. This insight deepens the understanding of the underlying mechanisms of disease co-occurrence and facilitates the development of precision treatments. The potential impact of this research extends beyond digestive cancers to other complex diseases with multiple comorbidities. Identifying distinct pathogenetic mechanisms underlying complex disease co-occurrence is crucial for the development of specific therapeutic strategies. This research contributes to the advancement of precision medicine and has the potential to impact public healthcare costs and improve quality of life for individuals affected by these conditions. 

## Data Availability

The phenotypic and genetic datasets of the UK Biobank used in this study are available through the UKB data access process. The application process for data access consists of six steps. For detailed information on how to apply for data access, one can refer to the following URL: https://www.ukbiobank.ac.uk/enable-your-research/apply-for-access (accessed on 1 March 2024). Detailed information about the data used in this study at the following URLs: http://www.ukbiobank.ac.uk/scientists-3/genetic-data/ (accessed on 1 March 2024) and http://biobank.ctsu.ox.ac.uk/crystal/label.cgi?id=100314 (accessed on 1 March 2024).

## Supplementary Materials

The following supporting information can be downloaded at: https://www.mdpi.com/article/10.3390/genes15040478/s1, Figure S1: The comorbidities for five digestive cancers. Column is ordered by correlation distance using hierarchical clustering using ‘Euclidean’ distance and “complete” method. “YES” indicates the disease is the comorbidity of the corresponding digestive cancer, vice versa; Figure S2: Overlapped potential susceptibility genes among five digestive cancers. Intersection size indicates the unique or shared susceptibility genes number for the digestive cancer(s) with black dot(s); Figure S3: Pleiotropic structure composition for the five digestive cancers. Y axis “Count” means the number of disease pairs. The number on the top of each bar means the proportion (the number of horizontal pleiotropic structure disease pairs divided by all pleiotropic structure disease pairs) within each digestive cancer; Table S1: Abbreviation, whole name and equivalent name for searching five digestive cancers and other diseases.; Table S2: Summary of Fisher’s exact test for each disease pair between five digestive cancers and other diseases.; Table S3: Summary of potential susceptibility genes deprived from three sources for five digestive cancers and other diseases.; Table S4: Pleiotropic genes, pathways, PPI, hub proteins and pleiotropic structure for each disease pair.; Table S5: GO gene-set enrichment scores of pleiotropic genes for each disease pair between five digestive cancers and other diseases. Table S6: The 150 diseases and corresponding 18 categories recruited in this study.

## Code Availability

We provide a Code Ocean [67] capsule: https://codeocean.com/capsule/0171413/tree (accessed on 1 March 2024) (including executable programming scripts, and input and output dat). The statistical analyses and plotting for this study were conducted using the following software and versions: RStudio version: 2022.12.0+353, R version: 4.1.2, and Bioconductor version: 3.14 (BiocManager 1.30.20).
