# Supplementary figures and images for: Integrated Pleiotropic Gene Set Unveils Comorbidity Insights across Digestive Cancers and Other Diseases
Source: Genes (Basel). 2024 Apr 10;15(4):478. doi: 10.3390/genes15040478 (PMC11049963; doi:10.3390/genes15040478)

# Digestive cancer

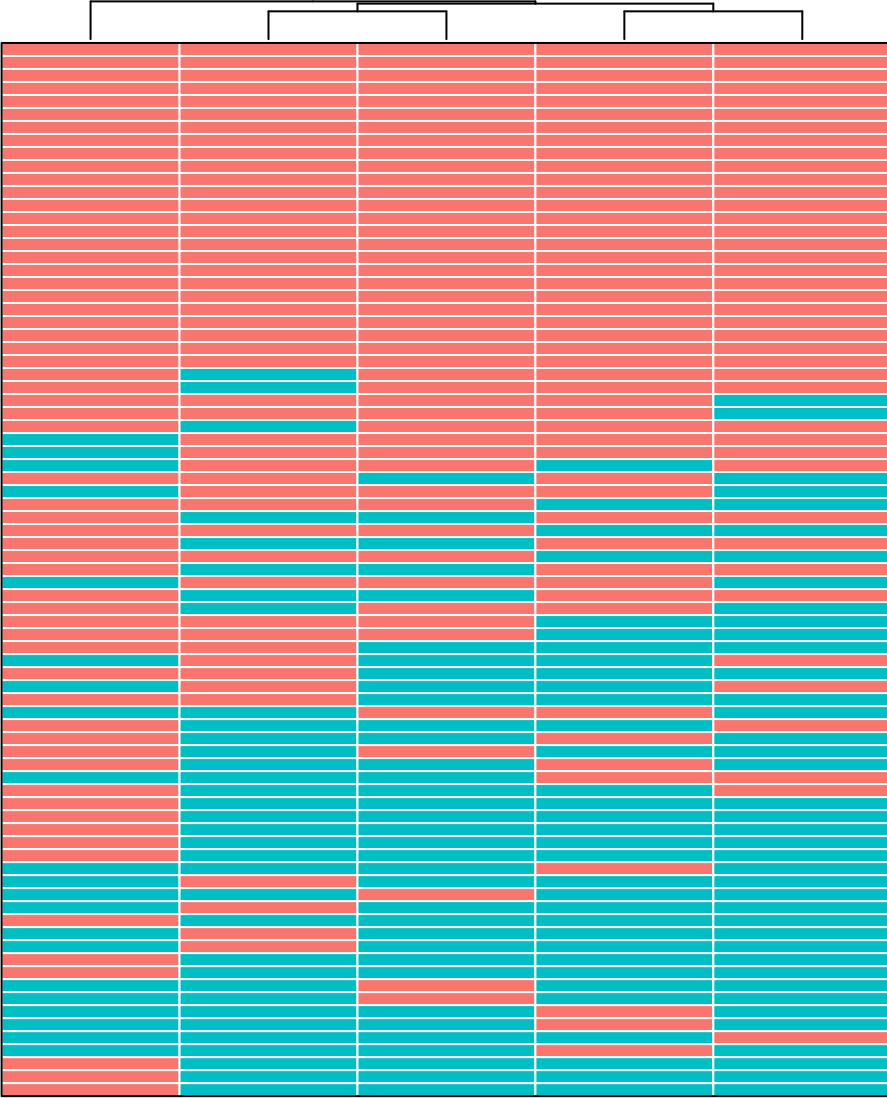

## Comorbidity

YES  
NO

Supplement: Supplementary file 1 [file genes-15-00478-s001.zip › Supplementary Figure S1.pdf]

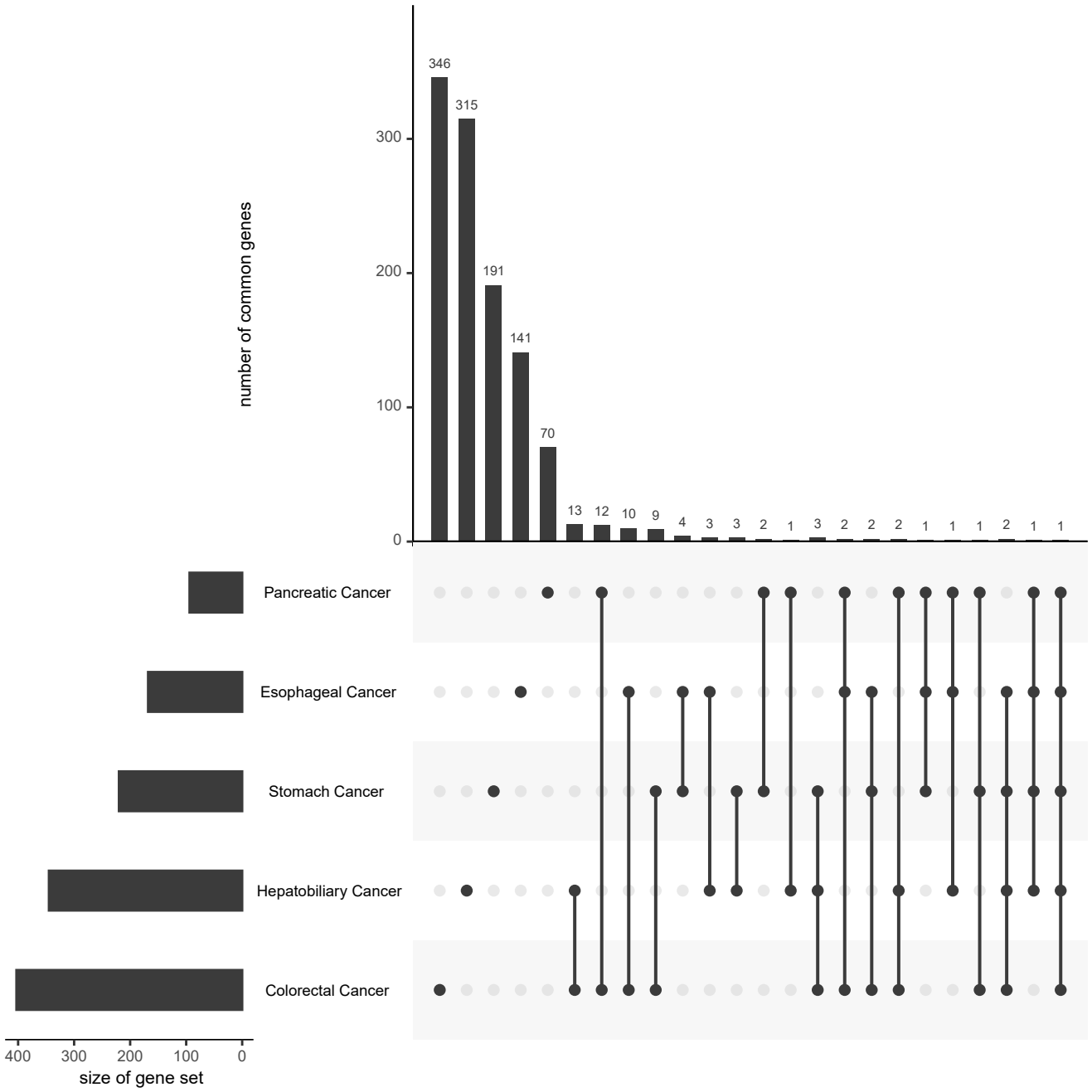

Supplement: Supplementary file 1 [file genes-15-00478-s001.zip › Supplementary Figure S2.pdf]

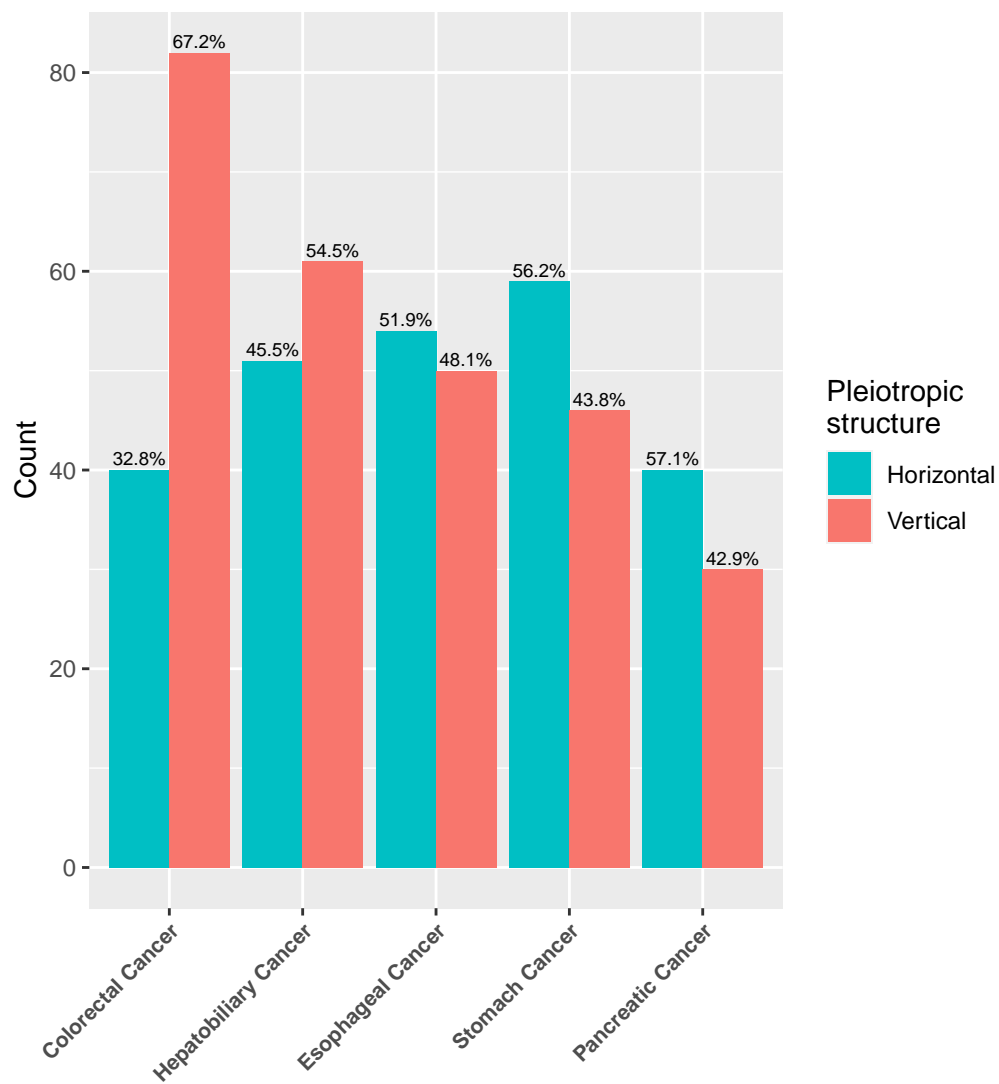

Supplement: Supplementary file 1 [file genes-15-00478-s001.zip › Supplementary Figure S3.pdf]
